# Fluid Biomarkers of Traumatic Brain Injury and Intended Context of Use

**DOI:** 10.3390/diagnostics6040037

**Published:** 2016-10-18

**Authors:** Tanya Bogoslovsky, Jessica Gill, Andreas Jeromin, Cora Davis, Ramon Diaz-Arrastia

**Affiliations:** 1 Center for Neuroscience and Regenerative Medicine, Uniformed Services University of the Health Sciences, Rockville, MD 20856, USA; cora.davis.ctr@usuhs.edu; 2 National Institute of Nursing Research, National Institutes of Health, Bethesda, MD 20814, USA; jessica.gill@nih.gov; 3 Quanterix Inc., Lexington, MA 02421, USA; ajeromin@quanterix.com; 4 Director, Traumatic Brain Injury Clinical Research Center, Department of Neurology, University of Pennsylvania Perelman School of Medicine, Philadelphia, PA 19104, USA; ramondia@mail.med.upenn.edu

**Keywords:** traumatic brain injury (TBI), biomarkers, TBI management

## Abstract

Traumatic brain injury (TBI) is one of the leading causes of death and disability around the world. The lack of validated biomarkers for TBI is a major impediment to developing effective therapies and improving clinical practice, as well as stimulating much work in this area. In this review, we focus on different settings of TBI management where blood or cerebrospinal fluid (CSF) biomarkers could be utilized for predicting clinically-relevant consequences and guiding management decisions. Requirements that the biomarker must fulfill differ based on the intended context of use (CoU). Specifically, we focus on fluid biomarkers in order to: (1) identify patients who may require acute neuroimaging (cranial computerized tomography (CT) or magnetic resonance imaging (MRI); (2) select patients at risk for secondary brain injury processes; (3) aid in counseling patients about their symptoms at discharge; (4) identify patients at risk for developing postconcussive syndrome (PCS), posttraumatic epilepsy (PTE) or chronic traumatic encephalopathy (CTE); (5) predict outcomes with respect to poor or good recovery; (6) inform counseling as to return to work (RTW) or to play. Despite significant advances already made from biomarker-based studies of TBI, there is an immediate need for further large-scale studies focused on identifying and innovating sensitive and reliable TBI biomarkers. These studies should be designed with the intended CoU in mind.

## 1. Introduction

TBI is an important public health concern, as it is one of the leading causes of death and disability around the world [1]. Recent epidemiological studies indicate that around 5.3 million people in the United States and nearly 7.7 million people in Europe live with a TBI-related disability [2]. Mild TBI (mTBI) accounts for 70%–90% of all cases [3]. Importantly, mTBI may be associated with persistent symptoms and prolonged disability in approximately 10%–20% of cases [4]. This indicates a significant public health issue due to the prevalence of TBI among civilians in the U.S. and around the world.

The ultimate challenge in the clinical practice of TBI resides in three major factors:

(1) Ambiguity in determining the clinical severity of TBI based on clinical variables, such as the Glasgow Coma Scale (GCS) [5], loss of consciousness (LOC) and posttraumatic amnesia (PTA). It is widely recognized that assessing TBI severity from GCS, LOC and PTA is imprecise [6]. Scores may be compromised in patients who are intubated or sedated, and GCS in acute settings has a low inter-rater reliability [7]. mTBI can especially be a challenge to diagnose, as the symptoms are often vague, inconsistent and overlap with other conditions that frequently confound the clinical picture, such as intoxication, delirium and functional disorders, like post-traumatic stress disorder (PTSD) [8]. The Abbreviated Injury Scale (AIS) is an anatomically-based, consensus-derived, global severity scoring system that classifies each injury by body region according to its relative importance on a six-point ordinal scale. Demetriades et al. evaluated the prognostic value of AIS by correlating head AIS with GCS using 7764 patients with head injuries. However, the study found that there was no good correlation (with correlation coefficient −0.31) between GCS and AIS [9].
(2) Relatively low sensitivity of the primary brain imaging modality used in the emergency department (ED), i.e., head computed tomography (CT) to identify subtle brain injuries, such as diffuse axonal injury (DAI) or microhemorrhages, which may significantly contribute to prolonged post-concussive symptoms and disability. Various studies have shown that only 10% of CT scans detect abnormalities in mTBI subjects [10,11]; whereas conventional MRI lacks the sensitivity to detect DAI (identifying approximately 32% of all DAI lesions) [12] and diffuse traumatic vascular injury (DVI), the most common abnormalities in mTBI [13,14]. Up to 30% of mTBI subjects had abnormalities in research MRI, including: microhemorrhages (identified by susceptibility weighted imaging (SWI) sequences), small contusions and edema (identified by diffuse weighted imaging (DWI) sequences) and meningeal enhancement (identified after intravenous contrast gadolinium administration on postcontrast fluid attenuated inversion recovery (FLAIR) images) [15]. These subtle and previously underdiagnosed types of brain injury have an important impact on the rate of recovery. The presence of DAI is associated with poor outcome after brain injury [16]. One study showed that DAI identified by MRI in the genu of the corpus callosum after TBI was associated with worse outcome after adjustment for age (*p* = 0.0051) [17]. Likewise, a large multicenter study demonstrated that the presence of ≥4 microhemorrhages after mTBI was associated with poorer three-month outcome with an odds ratio (O.R.) of 3.2 (*p* = 0.03) [15]. The visualization of DAI and DVI will likely require advanced methods, such as diffusion tensor imaging (DTI) and arterial spin labelling (ASL) (by demonstrating a decrease of cerebral flow in mTBI) [18], which are not routinely used in clinical practice. While MRI is safe, its application is limited for patients who are pregnant, claustrophobic, anxious, sedated, have cardiac pacemakers or retained metal shrapnel. MRI is also expensive, requires lying for a prolonged time in the scanner during image acquisition and cannot be tolerated by a subset of patients. Fluid biomarkers will likely be complementary to imaging and provide important tools for making progress in the management of TBI, particularly mTBI.
(3) Augmentation of pathophysiological changes in the chronic period, especially after repetitive TBI. Emerging evidence suggests that axonal loss and unique glial scarring after blast TBI [19] may be present for years post-injury. Furthermore, the consequences of repetitive injuries may not be accurately diagnosed by neuroimaging and may contribute to progressive neurodegeneration after TBI [20,21]. Elucidating the magnitude of axonal and vascular injury and persistent glial and axonal degeneration after TBI with the aid of fluid biomarkers may assist with accurate diagnoses and provide a link into possible therapies for these devastating conditions.

## 2. Pathophysiology of Biomarker Discoveries in TBI

TBI is an enormously diverse process that involves the interaction of numerous pathophysiological events and processes [16]. This presents a substantial challenge in determining reliable and sensitive biomarkers in TBI. TBIs result from a primary injury induced by an external force to the brain, such as direct impact, acceleration/deceleration or blast. The primary injury initiates a secondary pathophysiological cascade, which is characterized by excitotoxicity, the generation of free radicals and lipid peroxidation [22], mitochondrial dysfunction [23], swelling and loss of astrocytes [13], axonal swelling [24] and neuronal injury [25]. Secondary injury is associated with inflammatory response and alterations in both metabolism and cerebral blood flow [26], axonal lysis and breakdown with parenchymal accumulation of tau and amyloid beta (Aβ) protein [27], demyelination [28] and subsequent axonal degeneration [29] and programmed neuronal death through a caspase-3 activation mechanism [25]. Additionally, as a part of the healing processes, microglia proliferate then migrate to the site of injury and release cytokines, such as interleukin 1 (IL-1), interleukin-6 (IL-6) and tumor necrosis factor (TNF-α), which play dual roles by repairing and regenerating central nervous system (CNS) tissue, but may also be toxic to neurons and other glial cells [30]. 

One of the most established approaches to developing fluid biomarkers for TBI is identifying proteins abundant in brain cells, such as: neurons (neuron-specific enolase (NSE) [31], Ubiquitin C-terminal hydrolase-L1 (UCH-L1) [32,33], astroglia (S100B, glial fibrillary acidic protein (GFAP)) [31,33], oligodendrocytes (myelin basic protein (MBP)) [34], axonal cytoskeleton (tau [35,36], Aβ [37,38,39]), neurofilament light chain (NF-L) [40], phosphorylated neurofilament heavy chain (pNF-H) [41] and spectrin N-terminal fragment (SNTF) [42,43]. The second approach is to study inflammatory cytokines [44], metabolites and oxidized lipids [45] or to perform autoimmune profiling of novel TBI biomarkers associated with the pathophysiology of brain injury [46]. Numerous excellent reviews describing neuroinflammation after TBI have been recently published [47,48] and will not be reviewed here due to the limits of this manuscript. 

Below, we briefly outline the putative protein biomarkers reviewed in this paper.

Neuron-specific enolase (NSE) is an isoenzyme of enolase (2-phospho-d-glycerate hydrolase) located in the cytoplasm of neurons, which catalyzes the transition of 2-phosphoglycerate into phosphoenolpyruvate. NSE has been extensively studied as a blood biomarker of acute TBI [49,50].
S100B is a Ca-binding protein highly abundant in the astroglia of the brain. S100B is involved in the regulation of cell cycle progression and differentiation and has been extensively studied in TBI [51].
GFAP is a structural protein expressed almost exclusively in astrocytes and released upon disintegration of the cytoskeleton [52]. An astroglial injury marker, GFAP is found in both white and gray brain matter [53] and has been extensively studied as an indicator of intracranial pathologies. GFAP has been widely studied in TBI, and elevated levels of it in plasma show promise as a diagnostic and prognostic biomarker [54].
Ubiquitin C-terminal hydrolase-L1 (UCH-L1) [32] is a thiol protease that hydrolyzes a peptide bond at the C-terminal glycine of ubiquitin. UCH-L1 is a neuronal brain injury marker found in high abundance in the cytoplasm of neurons [55].
Myelin basic protein (MBP) is a major constituent of the myelin sheath of oligodendrocytes and Schwann cells in the nervous system. Acute disruptions of the myelin sheath were shown in experimental models, and acute myelin globoids were associated with axonal pathology in humans [28].
Neurofilaments (NF) are the key intermediate filaments in neurons. A major component of the axonal cytoskeleton, NF are an integral part of forming synapses and neurotransmissions [56]. The major neuronal intermediate filaments in the CNS are those assembled from the NF triplet proteins: neurofilament light (NF-L), medium (NF-M) and neurofilament heavy chain (NF-H). After a TBI and the resulting calcium influx, NF-H becomes phosphorylated (pNF-H), resulting in the accumulation of excessive dysfunctional pNF-H and a reduction in the integrity of the axons [56].
Tau is a microtubule-associated protein that acts as a structural element in the axonal cytoskeleton and is linked to axonal damage [57]. Widespread deposition of tau and the formation of neurofibrillary tangles (NFT) occurs following a single TBI or repetitive head injuries in CTE [27,58].
The amyloid-β_1–42_ (Aβ42) peptide is formed from the proteolytic cleavage of the amyloid precursor protein by β- and γ-secretases and rapidly aggregates to form oligomers, protofibrils and fibrils. This promotes the deposition of amyloid plaques associated with Alzheimer’s disease [59]. Accumulation of Aβ plaques in the human brain was found many years after a single TBI [27].
SNTF is a calpain-cleaved alpha II-spectrin N-terminal fragment and a newly-studied biomarker of neurodegeneration after TBI [60]. A recent study showed that SNTF immunoreactive axons were observed following both human severe and mTBI. Importantly, SNTF staining revealed a subpopulation of degenerating axons undetected by amyloid precursor protein (APP), the gold-standard marker of axonal transport interruption. [61]. SNTF protein released from degenerating axons after mTBI can be measured in CSF and in blood [42].

## 3. Definition of Biomarkers and Context of Use

Biomarkers have been historically critical to making progress in a broad range of clinical conditions [62,63]. To date, diagnostic and therapeutic advances in fields as diverse as cardiology, oncology, hematology and infectious diseases have depended on biomarkers as reliable and sensitive indicators to the underlying pathology [64,65]. A lack of similar biomarkers in the field of TBI is a major barrier to improving diagnostic evaluation and clinical care. There is a critical need for identifying, developing, and validating biomarkers to evaluate potential TBI diagnostics and treatments. According to a recent workshop on TBI Biomarkers by the Food and Drug Administration (FDA) “a biomarker is a defined characteristic that is measured as an indicator of normal biological processes, pathogenic processes, or responses to an exposure or intervention, including therapeutic interventions”. The FDA workshop emphasized the role of biomarkers in CoU by fully and clearly describing the way they are to be used and the medical product development-related purpose of the use. Biomarkers fall into four categories that are not mutually exclusive: diagnostic, prognostic, predictive and pharmacodynamic.

- Diagnostic biomarkers are disease characteristics that categorize persons by the presence or absence of a specific disease.
- Prognostic biomarkers are baseline measurements that categorize patients by degree of risk for disease progression and inform about the natural history of the disorder.
- Predictive biomarkers are baseline characteristics that categorize patients by their likelihood of response to a particular treatment.
- Pharmacodynamic biomarkers are dynamic measurements that show if a biological response has occurred in a patient after a therapeutic intervention [63].

In this review, the term “context of use” is conceptualized more broadly to encompass different settings where biomarkers could be useful in clinical research and eventually in clinical practice. Consideration of the CoU is important, as the particular purpose for which the biomarker is utilized greatly impacts issues related to the required specificity, sensitivity, as well as the analytical details. This review outlines the settings where diagnostic and prognostic biomarkers would be useful in clinical research and clinical practice. Our purpose is to outline the implications for the use of specific fluid biomarkers to aid with decision making in specific clinical settings. Our goal is to focus on the most studied fluid biomarkers, which may help to: (1) identify patients who may require acute neuroimaging (CT or MRI); (2) select patients at risk for secondary brain injury processes (e.g., increase of intracerebral pressure (ICP), hemorrhage growth, expansion of cerebral edema, ischemia or neuroinflammation); (3) aid in counseling with symptoms provided at discharge; (4) identify patients who are at risk for developing PCS, PTE or CTE; (5) predict outcome with respect to poor or good recovery; and (6) inform decisions regarding when to RTW or to play.

## 4. Development of Biomarker Assays from Body Fluids

Following TBI, biomarkers can be assayed primarily in CSF or peripheral blood [66]. CSF is in principle closer to brain tissue and is considered an optimal source for studying fluid biomarkers of TBI; yet, in less severe patients, there is a limit to the accessibility of CSF biomarkers [67]. However, the means of collecting CSF (i.e., lumbar puncture (LP) or ventriculostomy), as well as contaminated blood products can affect the accuracy of proteomic biomarker assays. Several CSF biomarkers of axonal injury (total tau, neurofilament light chain (NF-L)), and biomarkers of astroglial injury (S100B and GFAP) increase after severe and mild TBI [35,36,68]. On the other hand, mild and moderate TBI subjects, the most prevalent subgroup of the TBI population [69], do not have clinical indications for the collection of CSF. This fact limits the applicability of CSF biomarkers to clinical practice and concomitantly increases the importance of blood biomarkers for rapid and accurate diagnostic and prognostic assessments.

Studying blood-based biomarkers is challenging due to the low concentrations of the proteins of interest in the blood, their proteolytic degradation, clearance from the blood via liver or the kidney, binding to carrier proteins and the variable permeability of proteins to the blood brain barrier (BBB). The recently-discovered glymphatic pathway, which normally contributes to the clearance of interstitial proteins (including amyloid β and tau) from the brain, is impaired in TBI, a fact [70] that may further contribute to discrepancies between the levels of proteins in the brain and in the blood after the injury. The compatibility of blood biomarker studies is also affected by pre-analytical variabilities, which include the differences of assays in either plasma or serum, as well as the methods of sample preparation (e.g., contamination with platelets or with erythrocytes) and sample cryopreservation and thawing. Therefore, it is important to validate biomarker levels from samples collected in various hospitals and to analyze them in various laboratories. It is important to note that the low sensitivity of assays in plasma may hamper their diagnostic accuracy. For example, in many published studies, up to 25% of mTBI samples [54] have levels of GFAP and UCHL-1 below the low level of quantification (LLOQ), with a much higher rate of undetectable values in uninjured subjects. Likewise, previous studies revealed low sensitivity for the numbers of plasma assays in a healthy cohort and in mTBI [71]. Nevertheless, recent developments in assay technology indicate that several proteins that are both expressed in the brain and detectable in CSF can also be measured in lower concentrations in peripheral blood after TBI [35,72]. Furthermore, advances in novel assays to measure ultra-low concentrations of proteins in the blood along with measurements of the same biomarkers that indicate brain injury in CSF show promise and are rapidly progressing [38]. One example is the single molecule array (Simoa, Quanterix Corporation, Lexington, KY, USA) technology, which employs ultra-sensitive immunoassays and allows for accurate measurements of candidate biomarkers found at low concentrations in the blood [73,74]. For example, plasma tau is measurable by Simoa, which showed an increase of the protein in hockey players after concussion and in military personnel who sustained TBIs [74]. A recent study using Simoa showed increases in plasma tau and Aβ42 levels even up to 90 days after mild and moderate-to-severe TBI [75]. 

As mentioned above, TBI is an enormously complex process, and sensitive multiplex assays of potential fluid TBI biomarkers are currently under rigorous development. Recently, a multiplex analysis of 44 serum biomarkers in pediatric TBI showed that vascular cellular adhesion molecule (VCAM) was significantly decreased and IL-6 was increased, compared to controls [76]. Additionally, studies on autoimmune profiling showed that the antioxidant enzyme peroxiredoxin 6 (PRDX6) (which is highly expressed in brain astrocytes) may be a target for autoantibodies produced in response to TBI, as it is increased in TBI patients [46]. Furthermore, multiplexed assays of selected biomarkers (including S100B, GFAP, NSE, brain-derived neurotrophic factor (BDNF), monocyte chemoattractant protein-1, intercellular adhesion molecule (ICAM)-5 and PRDX-6) show promise in providing sensitive measurements of blood-based biomarkers in small volumes of blood [77].

An important challenge in the field of brain injury biomarkers is the lack of specific and sensitive biomarkers that may accurately distinguish TBI from the injury of peripheral tissues or from concomitant neurological and or/somatic diseases. For example, it is well established that the neuronal injury marker NSE is not specific to CNS injury, as it is increased after cardiopulmonary bypass, peripheral trauma, shock and ischemic reperfusion injury [50]. Furthermore, NSE is present in erythrocytes, blood platelets, plasmatic cells, lymphocytes, capillary walls and myoepithelial cells, which explains its physiologically low concentrations in peripheral blood. Likewise, protein S100B is found in peripheral Schwann cells, chondrocytes and adipocytes, in addition to astroglial cells [51]. Unsurprisingly, S100B is not specific to brain injury. It is increased after non-cranial trauma, myocardial infarction, as well as other pathologies and has significant variability with age and race [78]. Similarly, levels of glial marker GFAP and UCHL-1 were detected in patients with non-TBI injuries; however, concentrations of GFAP were significantly higher (*p* < 0.05) in TBI patients than in non-TBI trauma patients over seven days after injury, and concentrations of UCH-L1 were significantly higher in TBI patients than in orthopedic trauma patients up to 16 h post injury [33]. The presence of underdiagnosed concomitant pathologies of CNS or peripheral nerves may complicate the diagnostic value of fluid biomarkers of TBI. For example, axonal injury marker NF-H is increased in amyotrophic lateral sclerosis (ALS) as a marker of axonal degeneration of the anterior horn of the spinal cord [79], which may potentially contribute to increased NF-H levels after TBI. Another limitation of blood biomarkers is that they do not inform about the specific localization of the TBI. Spatial localization of the injury is important for predicting the patient’s outcome, making radiological examinations necessary. Overall, a careful clinical history of the patient’s concomitant diseases and other injuries, along with a clinical examination of brain trauma will help to correctly attribute increases in biomarkers when evaluating diagnostic, predictive and prognostic impacts of blood biomarkers in TBI. 

## 5. Context of Use of Blood Biomarkers of TBI

### 5.1. Pre-Hospital

The pre-hospital use of diagnostic or prognostic biomarkers through point-of-care devices has the potential to revolutionize TBI care when used at the scene of injury. Various point-of-care devices for biomarker assays are being developed for the purpose of informing emergency medical personnel or athletic trainers whether transport to an emergency department (ED) is needed or if the patient should be directly transported to a specialized neurosurgical center. Point-of-care tests are also important for military personnel who are deployed to remote stations and are at high risk for TBIs. Such biomarkers would need a very high level of sensitivity, with moderate specificity being sufficient. They would also have to be detectable in blood within minutes of injury by point-of-care tests. Applying these biomarkers to a pre-hospital setting will improve ED services by reducing unnecessary ambulance transport and ED evaluation. In addition, such point-of-care biomarkers would accelerate the transfer of patients to specialized neurosurgical centers, where such care can be lifesaving.

Point-of-care tests for TBI are currently under intensive development, with numerous field-usable biosensors capable of detecting multiplexed biomarkers of TBI within 2–30 min under way [80]. The current candidate biomarkers for point-of-care tests are primarily linked to astroglial or neuronal injury (GFAP, UCH-L1, NSE, S100B, tau and SNTF) [43,54,73,81], and it is likely that a combination of diagnostic biomarkers for point-of-care is warranted. 

#### 5.1.1. GFAP and UCH-L1

A recent study investigated the levels of UCH-L1 in 96 TBI patients with mild to moderate TBI (GCS 9–15) and 176 uninjured controls, with the average time from the injury to serum collection being 2.7 h [82]. Remarkably, UCH-L1 levels were detectable in this TBI group as early as 1 h after the injury. More specifically, UCH-L1 was able to differentiate TBI patients with a GCS score of 15 from uninjured control participants (area under curve (AUC) 0.87). That superior sensitivity and specificity for diagnosing TBI was obtained when GFAP was combined with UCH-L1 (AUC 0.94), thus supporting the idea that a combination of biomarkers may be superior compared to using each alone for the diagnosis and prognosis of TBI [54]. Recently, this study was confirmed on a larger cohort of TBI subjects in the hyperacute phase after TBI [33]. The investigators examined the diagnostic accuracy of GFAP and UCH-L1 separately and together in cohorts of mild and moderate TBI subjects (*n* = 584) with respect to the diagnostic precision of TBI, the presence of traumatic intracranial lesions detected by CT and the need for neurosurgical interventions. The level of GFAP peaked at 20 h and slowly declined, while UCH-L1 peaked at 8 h and declined over 48 h after mild and moderate TBI [33]. In conclusion, current data indicate that the early rise of UCH-L1 in combination with GFAP can be useful for detecting mTBI in hyperacute settings, including both civilian and military settings, and may be suitable for the development of point-of-care testing. 

#### 5.1.2. S100B

S100B has an excellent sensitivity to brain pathologies, but poor specificity, thus fulfilling the requirements needed for a TBI biomarker to be able to be utilized for prehospital use. S100B may serve as a potential point-of-care biomarker [51] despite significant observed age- and race-related variation in its accuracy for diagnosing mTBI. High levels of S100B protein have been linked to poor outcome following TBI and are correlated with injury severity [83]. In a study of 92 acute severe TBI (sTBI) patients (median admission GCS 6), levels of S100B were higher in non-survivors than in survivors [84]. S100B was lower in patients with focal lesions of <25 mL than in non-evacuated mass lesions and lower in swelling than in shifts of >0.5 cm. In 85 patients with sTBI, an increase of serum S100B concentrations of up to 1.13 ng/mL was associated with increased mortality (100% sensitivity; 41% specificity) and morbidity (88% sensitivity; 43% specificity) [85]. The American College of Emergency Physicians/Centers for Disease Control and Prevention states that in mTBI patients without significant extracranial injuries and serum S100B level less than 0.1 μg/L measured within 4 h of injury, consideration can be given not to perform a CT. Normal levels of S100B (if taken within 3 h of injury with the cutoff less than 0.10 μg/L) have been strongly correlated with the absence of CT-positive intracranial brain injury [86]. Likewise, recently-published Scandinavian guidelines use S100B for the screening of mTBI patients to select those who need cranial CT. Specifically, it was recommended that adult patients after mTBI with GCS 14 and no risk factors (anticoagulant therapy or coagulation disorders, post-traumatic seizures, clinical signs of depressed or basal skull fracture, focal neurological deficits) or GCS 15 with loss of consciousness or repeated (≥2) vomiting and no other risk factors will be sampled for analysis of S100B if less than 6 h have elapsed following trauma. The recommendations suggest that if S100B is less than 0.10 μg/L, the patient may be discharged without a CT [87]. However, measuring S100B as a biomarker is not an FDA-approved test for clinical use [88], and no biomarker measurements are currently recommended for the clinical use of TBI in adults [89].

#### 5.1.3. NSE

In a study of mTBI patients and 92 healthy controls, serum NSE (<6 h after TBI) was significantly elevated in mTBI patients compared to controls. The median NSE concentration was only slightly higher in TBI patients (9.8 mg/L; 10–90 percentile range 6.9–14.3 mg/L) than in controls (9.4 mg/L; 6.3–13.3 mg/L) and was not associated with any post-concussive symptoms [49]. Finally, in a study of concussed professional hockey players, changes of NSE were not significant in plasma and serum obtained before and after the season (median, 6.5 μg/L; range, 3.45–18.0 µg/L and 6.1 µg/L; range, 3.6–12.8 μg/L), correspondingly (*p* = 0.10) [73]. Therefore, compared to other biomarkers of mTBI, the importance of NSE for rapid accurate diagnostics, especially of mTBI, may be limited. 

In conclusion, a number of promising candidates exist for point-of-care devices, which warrants their rapid development and implementation into practice. Specifically: (1) serum GFAP and UCH-L1 increases 1 h after TBI, with peaks at 20 h and 8 h, respectively, which makes them very attractive biomarker candidates; (2) a combination of GFAP with UCHL-1 may represent a more sensitive marker of TBI than each of them separately; (3) due to the excellent sensitivity, but poor specificity, low serum S100B levels in the first few hours following injury, when combined with other diagnostic measures, may help to reduce the number of unnecessary CT scans; (4) while serum NSE may be elevated in mTBI, its low specificity to brain trauma and presence in red blood cells makes it difficult to utilize NSE for accurate diagnostics of mTBI in point-of-care devices.

### 5.2. Emergency Department

Diagnostic and prognostic biomarkers would be useful in the ED for several clinical indications:

#### 5.2.1. Identifying Patients in Need of Cranial CT Scanning

CT is very effective at identifying lesions (such as acute epidural hematomas (EDH), worsening of contusions, hemorrhages, cerebral edema, subdural hematomas (SDH) and hydrocephalus) that may be life threatening or with the potential to progress over time [90]. However, as has been pointed out by many recent observers [88], it is possible that cranial CT is over-utilized in the evaluation of TBI, which may result in substantial expenses and potentially dangerous radiation exposure. A biomarker that would inform the clinical decision to obtain or defer a cranial CT would have a major economic impact, as well as reduce harmful radiation exposure. In order to be useful, this type of biomarker would need a very high level of sensitivity, but only moderate specificity. 

##### GFAP and UCH-L1

In an mTBI population, Metting and colleagues demonstrated that serum GFAP was increased in patients with an abnormal CT, compared with those with a normal CT [91]. Similarly, Papa and colleagues found that serum GFAP was significantly higher in mTBI patients with intracranial lesions on CT, compared with those without lesions and predicted patients who required neurosurgical intervention [82]. A recent study evaluated temporal profiles of GFAP and UCH-L1 in a large cohort of mild to moderate TBI patients and showed that GFAP outperformed UCH-L1 in accuracy for detecting intracranial lesions on CT. This was based on the findings that the diagnostic range of AUC’s for GFAP was higher (0.80–0.97), when for UCH-L1, the AUC was lower (0.31–0.77) [33]. Another study evaluated neuronal injury marker UCH-L1 and GFAP from 324 consecutive patients with acute TBI (mild 57%, moderate 12% and severe 31% by the admission GCS) and 81 control subjects. The baseline measures included head CT scanning with Marshall Grade evaluation and blood sampling. In line with previous studies, both biomarkers in plasma were capable of distinguishing mass lesions (40% of CT) from diffuse injuries in CT (20%) assessed by the Marshall Grade [92]. 

##### Neurofilaments

As pointed out earlier, DAI is the signature injury in TBI [13,24,29]. Likewise, DAI was recently identified by the presence of APP-positive axonal swellings typical after blast exposure [93]. NF are structural components of axons. Following TBI, peripheral elevations of NF-L chain and pNF­H are considered to be an axonal injury marker [67]. Gatson et al. [94] studied serum pNF­H in patients with mTBI (*n* = 34, admission GCS 13–15) at Days 1 and 3 after the injury. Receiver operator analysis (ROC) showed that the AUC for the CT-positive TBI group vs. the CT-negative group was significant (*p* = 0.021) with a sensitivity of 87% and a specificity of 70% using a cutoff of 1071 pg/mL at Day 1. These results suggest that elevated levels of serum pNFL-H in ED settings may be useful in determining which individuals require CT imaging by assessing the severity of their injury. 

#### 5.2.2. Informed Counseling Provided at ED Discharge

PCS is a clinical entity referred to as the presence of persistent neurological symptoms lasting for more than three months and is observed in 40%–80% of individuals exposed to mTBI [95]. It is a potentially debilitating syndrome that consists of physical symptoms (headache, dizziness, fatigue), cognitive disturbances (impaired concentration and memory) or emotional problems, including depression and anxiety. About 10%–15% of individuals with TBI experience persistent symptoms after one year [96]. PCS itself is difficult to diagnose as symptoms overlap with other disorders that can occur independently of brain injury, such as depression, substance abuse and post-traumatic stress disorder (PTSD). 

Recent studies have pointed out the overall poor level of counseling for many patients with mTBI provided at the time of ED discharge [97]. Many patients with TBI who have negative cranial CT scans are not properly counseled on how their PCS is expected to evolve, resulting in premature attempts to RTW, school or other regular activities. This results in unnecessary repeat visits to the ED and (potentially) the underutilization of rehabilitative services. RTW [98] after TBI may be a challenge because of possible physical, cognitive and emotional impairments, but is important as a major indicator of real-world functioning. Furthermore, current evidence concerning the duration of symptoms of sports concussion and its prognosis is very preliminary, and there is no evidence on the effect of return-to-play guidelines on the prognosis of TBI [99]. A diagnostic or prognostic biomarker that helps to inform ED staff on the nature of the brain injury and, therefore, the expected trajectory of recovery would improve their ability to counsel patients and provide them with more realistic expectations for recovery. 

##### S100B

As mentioned above, astroglial marker S100B is extensively studied as a prognostic and predictive biomarker of TBI. Serum S100B was inversely associated with rates of RTW in 93 patients after very mild TBI (admission GCS 15, either with or /without LOC and/or PTA). In this study, an increase of serum S100B (>0.15 μg/L) was associated with a failure to RTW within one week after the injury. The inability of mTBI patients with elevated S100B to RTW was 37.5%, versus 4.9% in those with normal values. Furthermore, patients with S100B above 0.15 μg/L had an 11-fold probability of failure in their short-term RTW or activities [100]. 

Associations of fluid biomarkers with various postconcussive symptoms have also been extensively examined. A study in mTBI patients evaluated the association between increases in serum S100B 6 h after the injury and symptoms of headache, nausea, vomiting and dizziness. The study found that patients who vomited had a higher median S100B concentration compared to patients who did not (0.5 vs. 0.25 mg/L; *p* = 0.03). Patients with headaches, however, had a lower median S100B concentration than patients without headaches (0.21 vs. 0.33 mg/L; *p* = 0.02). However, there was no association between S100B and the symptoms of nausea and dizziness [49]. 

##### Tau

Previously-used assays for tau are adequate for CSF, but are not sensitive enough to reliably detect low concentrations found in blood [37]. Recent studies using the highly sensitive Simoa assay indicate that sports-related TBIs are associated with increases of tau in plasma [35]. Shahim et al. showed that total tau levels in plasma were increased in concussed players compared to pre-season levels. It is important to note that the highest tau levels were measured immediately after the concussion (1 h after the concussion), but they decreased during rehabilitation. Surprisingly, the duration of post-concussion symptoms was correlated with tau levels measured 1 h after concussion (*r*^2^ = 0.34; *p* = 0.002) [73]. Therefore, plasma tau may be useful for counseling patients about the duration of PCS and RTW or to play after TBI.

##### Neurofilament Light Chain

NF-L, as previously mentioned, has emerged as a promising biomarker for neurological disability. In 30 amateur boxers who experienced concussions following boxing activity (a minimum of 45 bouts within 1–6 days), NF-L levels were increased in CSF, both after a bout, as well as after a rest period of at least 14 days (532 ± 553 vs. 135 ± 51 ng/L, *p* = 0.001). The researchers concluded that the lack of normalization of NF-L after rest may indicate ongoing brain degeneration [35]. Another study evaluated the levels of NF-L in CSF and serum in TBI patients (*n* = 182) and found an increase of serum NF-L on Days 1–15 after the TBI, which correlated with the outcome of Glasgow Outcome Scale (GOS) six or 12 months later. This study found a correlation between levels of NF-L obtained in CSF through ventriculostomy and in peripheral blood [40]. Therefore, higher levels of NF-L in TBI patients (those with ongoing secondary neurodegeneration after TBI and after repetitive TBI) may indicate a need for further counseling, follow up, or an extended rest period after trauma. 

##### SNTF

Increased levels of newly-developed axonal injury marker SNTF on the day of the mTBI correlated significantly with cognitive impairment persisting for at least three months. In this study, SNTF on the day of mTBI showed 100% sensitivity and 75% specificity for predicting failure to improve cognitive performance over the first three months after a CT-negative mTBI [42]. Recently, serum SNTF was studied in professional ice hockey players as a biomarker of sport-related concussions. SNTF was able to differentiate the severity of concussion and to predict return to play. Specifically, the study showed that in 20 players withheld from play for six days or longer, serum SNTF levels rose 1 h to six days after a concussion and differed significantly from the less severe concussions (*p* = 0.004). In addition, serum SNTF showed good diagnostic accuracy for delayed return to play (AUC 0.87) [43].

In summary: (1) an increase in serum pNF-H measured in the ED setting is able to distinguish patients with mTBI who may need CT to determine intracranial injury from those who do not need imaging; (2) an increase in plasma tau 1 h after injury correlates with the duration of PCS and may aid in counseling regarding RTW or to play after TBI; (3) increased levels of serum NF-H after TBI may be associated with cognitive problems; (4) a high S100B concentration after mTBI may be associated with symptoms after TBI, such as vomiting; (5) high serum S100B (>0.15 μg/L) after mTBI may indicate delayed RTW; (6) increased plasma SNTF on the day of injury correlated significantly with cognitive impairment.

### 5.3. Intensive Care Unit

#### 5.3.1. Early Detection of Secondary Brain Injury

The focus of neurological ICU care for the past several decades has been the prevention of secondary brain injury, from factors such as ischemia, hypoxia, edema, inflammation or intracranial hypertension. While such efforts have overall been a success (the case fatality rate for sTBI has fallen from approximately 40% in the 1980s to under 20%) [101], no specific biomarker of early secondary injury is currently available. Identifying such a biomarker is important, as this is a time period in which these processes are potentially reversible, supporting the need to fully validate and fully adopt such a biomarker. Such a prognostic biomarker would require high sensitivity and specificity.

##### S100B

Serial sampling of S100B could detect secondary deterioration in unconscious patients including TBI. Raabe et al. [102] studied serial blood S100B levels in 246 neurocritical care patients, including 31 patients with sTBI. The study indicated that a neurological deterioration occurred in 33 patients (13%), while all patients had increased serum S100B values (mean 2.00 μg/L, standard deviation 2.61 μg/L, range 0.31–9.66 μg/L). In the case of sedated and intubated sTBI patients, an increase of S100B on the 13th day (up to 1.99 μg/L) was associated with ischemic complication, later confirmed by emergency CT, which otherwise may not have been recognized [102]. A retrospective study on 250 sTBI patients showed that secondary increases in serum levels of S100B, even as low as ≥0.05 μg/L, beyond 48 h after TBI were strongly correlated with the development of clinically-significant secondary CT/MRI findings (including cerebral infarction, edema, hematoma, herniation and sinus thrombosis) [103].

##### Markers of Neuroinflammation

As mentioned above, the secondary insults after TBI involve an exceptionally complex interplay of numerous factors and substances, including alterations in cerebral blood flow, biochemical derangements, edema, oxidative stress, release of excitotoxic mediators, inflammation, apoptosis and necrosis [16]. It should be noted that TBI also compromises the integrity of the BBB, which contributes to the propagation of vasogenic and cytotoxic edema and allows the infiltration of inflammatory cytokines and chemokines into the brain parenchyma, thereby promoting the infiltration of inflammatory cells. It is well recognized that these secondary insults contribute significantly to the outcome [104]. Neuroinflammation is an important feature of secondary injury [105]. The role of cytokines in neuroinflammation after TBI has been studied extensively. In particular, increases of interleukin (IL)-1β, IL-6, the chemokine IL-8, IL-10 and (TNF)-α have been noted in both the serum and CSF of patients with TBI [105,106].

Intracranial hypertension (ICH) and cerebral hypoperfusion (CH) are well-known serious consequences of sTBI, which have a negative influence on the outcome, with no reliable methods of clinical prediction currently available. A recent study [107] evaluated levels of IL-1β, IL-6, IL-8, IL-10 and (TNF)-α in both serum and CSF, then related these biomarkers with hourly values for intracranial pressure (ICP) and cerebral perfusion pressure (CPP) in patients with sTBI (*n* = 24, admission GCS < 9). The cumulative pressure time dose (PTD; mm Hg·h) for ICP > 20 mm Hg (PTD ICP20) and CPP < 60 mm Hg (PTD CPP60) was compared with the serum and CSF levels. The study found that the PTD ICP20 and PTD CPP60 were moderately correlated with increased IL-8 levels (*r* = 0.34, *p* < 0.001; *r* = 0.53, *p* < 0.001). Therefore, it is plausible that increases in IL-8 may predict impending secondary injury, such as ICH and CH, before their clinical manifestation occurs.

#### 5.3.2. Inform Decisions to Withhold or Withdraw Care

Almost all clinicians believe that delivering futile care, which may prevent death, but result in permanent minimally-conscious states, is unethical and against the wishes of most patients and families. Unfortunately, these decisions are currently made very inconsistently from hospital to hospital and even among different clinicians in the same hospital. A prognostic biomarker that would increase the accuracy of such predictions would inform both clinicians and families on these critical decisions. The decision to withhold clinical care is usually based on complex clinical, laboratory and radiological evaluations. It has been pointed out that the prognostic models used in this process should have strong discriminative power [108]. Few prognostic models for TBI outcomes have been proposed. One model (the CRASH (corticosteroid randomization after significant head injury) CT) assessed age, level of consciousness (GCS), pupil reactivity, presence of major extracranial injury and findings on cranial CT within 8 h after injury. This model predicted: (a) death at 14 days with AUC 0.71–0.87; and (b) unfavorable outcome at six months (GOS 1–3) with AUC 0.71 [109]. Another model (IMPACT (International Mission for Prognosis and Analysis of Clinical Trials in TBI) extended model) included age, motor score, pupillary reactivity, hypoxia, hypotension, CT characteristics, glucose and hemoglobin assessed within the first few hours after the injury. This model predicted: (a) unfavorable outcome (GOS 1–3) at six months with AUC 0.71–0.86; and (b) mortality at six months with AUC 0.71 [110]. However, as previously mentioned, prognostic models are not absolutely accurate to serve as the sole basis for deciding to limit treatment [108]. Using biomarkers to assist with withholding care especially in the acute period after TBI should be considered with caution.

##### S100B

A recent study investigated the prognostic value of S100B for the outcome prediction of patients with sTBI. The study found that serum concentrations of S100B higher than 0.2 μg/L on Day 1 were associated with unfavorable outcome (GOS 1–3) with the O.R. for a worse outcome being 7.6 (95% confidence interval (CI) 2.25–25.80 *p* = 0.001) compared to patients with a favorable outcome (GOS 4–5). Most importantly, they reported that an S100B serum concentration of 0.7 μg/L on Day 1 correlated with 100% mortality [111]. However, in a retrospective study of 265 TBI patients, Thelin et al. showed that early samples of S100B within 12 h after injury may have limited prognostic value [112].

Elevated GFAP, S100B and NSE levels are associated with increased mortality after sTBI. In a study that enrolled 85 patients with sTBI (admission GCS < 8), GFAP levels were elevated in CSF and serum, particularly in patients who experienced unfavorable outcome, which was assessed six months later [85]. Compared to controls, the median serum increase in these patients was 4.6-fold for GFAP and two-fold for NSE. GFAP had the highest predictive value, whereas S100B and NSE less strongly predicted poor outcome (with adjusted O.R. 8.82 (GFAP), 5.12 (S100B) and 3.95 (NSE), respectively).

##### Aβ42

Another potential candidate for outcome prediction is Aβ42. A study on 12 TBI patients with median GCS 7 (range 3–8) showed that an increase of plasma Aβ42 was significantly higher in non-survivors (GOS 1, measured six months after the injury) than in survivors (GOS 5–8) (27.97 pg/mL (IQR (Interquartile rate), 13.66–32.90 pg/mL) vs. 16.29 (14.13–18.88 pg/mL)), correspondingly (*p* < 0.0001) [38]. The same study showed that, compared to the control levels, plasma Aβ42 levels were increased up to seven days after the TBI with its peak on Day 6 [38].

##### BBB Injury Markers

Injury to the BBB following TBI may be associated with the development of brain edema. This leads to an expansion of brain volume that has a crucial impact on morbidity and mortality due to an increase of ICP, impairment of CPP, oxygenation, and ischemic injuries. Matrix metalloproteinase-9 (MMP-9) and cellular fibronectin (cFn) are markers of BBB integrity, and in experimental studies, their increase is associated with impairment of BBB function after TBI [113]. In patients with sTBI, MMP-9 and cFn plasma concentrations assessed 6, 12, 24 and 48 h after injury predicted both the length of stay in the ICU and likelihood of death [114]. 

To conclude: (1) increased levels of IL-8 and TNF-α in CSF may predict impending secondary injuries, such as ICH and CH, before their clinical manifestation; (2) increases of plasma GFAP, and S100B (5–8-folds above reference levels) may correlate with poor outcome; (3) an increase in S100B serum concentration (of 0.7 μg/L) predicts 100% mortality; (4) increased plasma Aβ42 levels (up to 27.97 pg/mL) may predict poor outcome; (5) increased MMP-9 and cFn may predict poor outcome. 

### 5.4. Rehabilitation Unit

While there has been prior work on biomarkers in the preclinical, ED and ICU settings, there are essentially no prior findings reporting on biomarkers relevant in the rehabilitation unit. This is an important knowledge gap, as prognostic, predictive and pharmacodynamic biomarkers would be useful in the rehabilitation unit for several purposes. 

A recent study analyzed plasma GFAP, tau and Aβ42 from 34 subjects with mTBI and moderate-to-severe TBI collected within 24 h (Day 0), 30 and 90 days after injury. The study found that GFAP, tau and Aβ42 were increased up to 90 days after TBI compared to controls. The levels of GFAP and tau were maximal at Day 0 and at Day 30 for Aβ42. Day 30 Aβ42 correlated with GOSE (Glasgow outcome scale extended) (*p* = 0.042) when assessed six months after injury [75]. In addition, late predictors of outcome in sTBI were evaluated on a sample of 107 patients with sTBI (admission GCS 4–8) [115]. An unfavorable GOS score (1–3) at one year was predicted by higher Day 7 GFAP levels (above 9.50 ng/mL; AUC 0.82). Non-survivors at one year had significantly higher Day 7 GFAP serum levels (above 11.14 ng/mL; AUC 0.81) and Day 7 IL-6 serum levels (above 71.26 pg/mL; AUC 0.87). These findings support that GFAP and IL-6 monitoring could aid in prognosticating outcomes in patients with subacute sTBI.

To conclude: (1) increases of Day 7 GFAP levels (above 11.14 ng/mL) and Day 7 IL-6 serum levels (above 71.26 pg/mL) may predict worse outcome; (2) plasma GFAP, tau and Aβ42 may be increased up to 90 days after moderate to severe and even mTBI.

### 5.5. Chronic Phase

Since TBI exposure has been associated with long-term neurodegeneration, the latent effects of TBI are an important and understudied issue. While cognitive deficits improve within three months of injury, it is estimated that 5%–15% of all mTBI patients suffer persistent symptoms for months to years after the injury preventing their RTW [116]. Predictors of delayed RTW after mTBI include a lower level of education, nausea or vomiting on hospital admission, extracranial injuries, severe head/bodily pain early after injury and limited job independence and decision-making latitude [98]. Another study found that age, multiple bodily injuries, intracranial abnormality at the day of injury and fatigue predicted slower RTW after mTBI [117]. To date, limited data are available on biomarkers during the subacute (8–90 days) and chronic (>90 days) periods after TBI to aid with RTW prediction.

Plasma tau was increased in military personnel who sustained TBI (incurred up to 18 months before the clinical and laboratory evaluation) compared to healthy controls (mean (SD) 1.13 (0.78) vs. 0.63 (0.48) pg/mL, correspondingly). Having three or more TBI was associated with higher levels of tau than having only one with mean (SD) 1.52 (0.82) vs. 0.82 (0.60 pg/mL). Self-reported symptoms of postconcussive disorder were determined by the Neurobehavioral Symptom Inventory; the study showed a correlation between tau and the severity of post-concussive symptoms (*r* = 0.37; *p* = 0.003) [74].

#### 5.5.1. Identification of Patients at Risk for Post-Traumatic Dementia

Epidemiologic studies over the past decade have identified that individuals who suffer TBI in early and mid-life are at increased risk of developing dementia in later life. The increased relative risk is modest (in the order of 1.5–2-fold), but since the population risk of late in life dementia is so high, the absolute number of patients affected is very high. It is estimated that approximately 10% of the population-attributable risk of late in life dementia is due to TBI [118].

Further, tau pathology is a prominent finding in post-mortem assessments of boxers, American football players, military personnel and others who have suffered repetitive concussive traumatic brain injuries [20,58]. In CTE, the pathognomonic finding is an abnormal perivascular accumulation of hyperphosphorylated tau (p-tau) as neurofibrillary tangles, astrocytic inclusions and neurites, distributed at the depths of the cortical sulci. At this time, CTE can only be diagnosed by neuropathological examination. Recently, a promising serum biomarker has been identified by measuring the serum levels of the neuronal protein tau. Exosomes are nanovesicles released by most cells throughout the body, including the brain, into the extracellular environment through exocytosis of plasma membrane-anchored vesicles. The molecular content or cargo of exosomes directly reflects the content of the cell of origin. A recent study examined tau-positive exosomes in plasma as a potential CTE biomarker in 78 former National Football League (NFL) players and 16 controls. To this end, extracellular vesicles were isolated from plasma, and fluorescent nanoparticle tracking analysis was used to determine the number of vesicles staining positive for tau. The study found that the NFL group had higher exosomal tau than the control group (*p* < 0.0001). Exosomal tau discriminated between the groups, with 82% sensitivity, 100% specificity, 100% positive predictive value and 53% negative predictive value. Of note, within the NFL group, higher exosomal tau was associated with worse performance on tests of memory (*p* = 0.0126) and psychomotor speed (*p* = 0.0093) [119].

#### 5.5.2. Identification of Patients at Risk of Post-Traumatic Epilepsy

PTE is one of the best studied chronic effects of neurotrauma. This risk is approximately 4–20-fold and four-fold greater than the general population risk of epilepsy, respectively [120]. The risk of PTE after mTBI is lower, probably in the order of 1.3-fold, yet this risk is still considerable [121]. Following TBI, there is a variability in the time in which the first seizure occurs, with clinical onset reported >10 years post-injury [120]. Therefore, developing antiepileptogenic therapies to prevent PTE in those patients most at risk through biomarkers, specifically those that relate to chronic inflammatory and aberrant synaptic plasticity, will provide insights into the mechanism of PTE following a TBI. 

Growing evidence associates glial cell activation and subsequent cytokine production following acute seizures as an important contributor to epileptogenesis, which is highly relevant to TBI. A similar glial cell and cytokine response exhibited at epileptogenesis is also observed following TBI. One of the most widely-studied biomarkers for epileptogenesis is IL-1β, a proinflammatory cytokine produced in the CNS by activated microglia and astrocytes. Increased IL-1β production following TBI increases CNS hyperexcitability and excitotoxicity through Ca2+, glutamatergic and GABA-ergic mechanisms, potentially contributing to epileptogenesis [122]. 

A recent study assessed genetic variation in the IL-1β gene, finding both that Il-1β levels in CSF and serum and CSF/serum IL-1β ratios are related to the risk for the development of PTE. With this in mind, 256 adults with moderate-to-severe TBI were followed to investigate the development of PTE. In these subjects, IL-1β tagging and functional single nucleotide polymorphisms (SNPs) were genotyped. Genetic variance and PTE development was assessed. Serum and CSF IL-1β levels were collected from a subset of subjects (*n* = 59) during the first week post-injury and were evaluated for their associations with IL-1β gene variants and also PTE. The study found that higher CSF/serum IL-1β ratios were associated with increased risk for PTE over time (*p* = 0.008). Multivariate analysis for rs1143634 revealed an association between the CT genotype and increased PTE risk over time (*p* = 0.005). The CT genotype group also had lower serum IL-1β levels (*p* = 0.014) and higher IL-1β CSF/serum ratios (*p* = 0.093). Therefore, the findings of this study implicated IL-1β gene variability in PTE risk and linked IL-1β gene variation with the serum IL-1β levels observed after TBI. It also associated IL-1β ratios with risk of PTE [123].

To conclude: (1) plasma tau may be increased in chronic TBI patients who have multiple concussions and post-concussion symptoms; (2) exosome tau isolated from plasma may be increased in patients with repetitive concussions and associated with decreased memory and psychomotor performance; (3) measurements of exosome tau in plasma may, after additional future research, prove to be an accurate, noninvasive biomarker for CTE; (4) IL-1β gene variation and serum IL-1β levels may be linked to an increased risk of PTE. However, these promising pilot findings warrant future validations.

## 6. Future Perspectives

The search for TBI biomarkers is exceptionally challenging due to the tremendously complex processes of numerous pathophysiological events taking place after brain injury. While CSF biomarkers may reliably reflect biochemical and physiological changes in the brain the development of ultrasensitive assays is warranted, as most TBIs are moderate or mild and are reliant on peripheral blood samples. It is plausible that serial samples of blood biomarkers will allow for rapid diagnosis, the monitoring of a patient’s well-being, and improvement in their outcomes during future pharmacological treatments of TBI. To develop reliable diagnostic, prognostic, predictive and pharmacodynamic blood biomarkers of TBI, large-scale multicenter studies involving thousands of patients and careful clinical assessment with advanced neuroimaging techniques will likely be required to address this critical issue.

## Abbreviations

TBI	Traumatic brain injury
CSF	Cerebrospinal fluid
CT	Cranial computerized tomography
MRI	Magnetic resonance imaging
PCS	Postconcussive syndrome
PE	Posttraumatic epilepsy
CTE	Chronic traumatic encephalopathy
mTBI	Mild TBI
GCS	Glasgow Coma Scale
LOC	Loss of consciousness
PTA	Post-traumatic amnesia
PTSD	Post-traumatic stress disorder
ER	Emergency room
DAI	Diffuse axonal injury
DVI	Diffuse traumatic vascular injury
SWI	Susceptibility weighted imaging
DWI	Diffuse on weighted imaging
FLAIR	Fluid attenuated inversion recovery
O.R.	Odds ratio
DTI	Diffusion tensor imaging
ASL	Arterial spin labeling
CNS	Central nervous system
NSE	Neuron specific enolase
UCH-L1	Ubiquitin C-terminal hydroase-L1
GFAP	Glial fibrillary acidic protein
MBP	Myelin basic protein
NF-L	Neurofilament light chain
pNF-H	Phosphorylated neurofilament heavy chain
NF-M	Neurofilament medium chain
NFT	Neurofibrillary tangles
Aβ42	Amyloid-β 42
SNTF	Spectrin N-terminal fragment
APP	Amyloid precursor protein
CoU	Context of use
FDA	Food and Drug Administration
ICP	Intracerebral pressure
LP	Lumbar puncture
BBB	Blood brain barrier
LLOQ	Low level of quantification
Simoa	Single molecule array
VCAM	Vascular cellular adhesion molecule
PRDX6	Peroxiredoxin 6
BDNF	Brain-derived neurotrophic factor
(MCP-)1	Monocyte chemoattractant protein
(ICAM)-5	Intercellular adhesion molecule
ALS	Amyotrophic lateral sclerosis
ED	Emergency department
AUC	Area under curve
sTBI	Severe TBI
EDH	Epidural hematomas
SDH	Subdural hematomas
ROC	Receiver operator analysis
RTW	Return to work
Aβ	Amyloid beta
TNF	α-Tumor necrosis factor
ICH	Intracranial hypertension
CH	Cerebral hypoperfusion
CPP	Cerebral perfusion pressure
PTD	Pressure time dose
CI	Confidence interval
MMP-9	Matrix metalloproteinase-9
cFn	Cellular fibronectin
NFL	National Football League
PTE	Posttraumatic epilepsy
SNPs	Single nucleotide polymorphisms
